# The relationship between adipokine levels and bone mass—A systematic review

**DOI:** 10.1002/edm2.408

**Published:** 2023-02-09

**Authors:** Darren Mangion, Nikolai P. Pace, Melissa M. Formosa

**Affiliations:** ^1^ Department of Applied Biomedical Science, Faculty of Health Sciences University of Malta Msida Malta; ^2^ Centre for Molecular Medicine and Biobanking University of Malta Msida Malta; ^3^ Department of Anatomy, Faculty of Medicine and Surgery University of Malta Msida Malta

**Keywords:** adipokines, adiponectin, bone mineral density, diabetes, leptin, osteoporosis

## Abstract

**Introduction:**

Adipose tissue is the source of a broad array of signalling molecules (adipokines), which mediate interorgan communication and regulate metabolic homeostasis. Alterations in adipokine levels have been causally implicated in various metabolic disorders, including changes in bone mass. Osteoporosis is the commonest progressive metabolic bone disease, characterized by elevated risk of fragility fractures as a result of a reduced bone mass and microarchitectural deterioration. The effects of different adipokines on bone mass have been studied in an attempt to identify novel modulators of bone mass or diagnostic biomarkers of osteoporosis.

**Methods:**

In this review, we sought to aggregate and assess evidence from independent studies that quantify specific adipokines and their effect on bone mineral density (BMD).

**Results:**

A literature search identified 57 articles that explored associations between different adipokines and BMD. Adiponectin and leptin were the most frequently studied adipokines, with most studies demonstrating that adiponectin levels are associated with decreased BMD at the lumbar spine and femoral neck. Conversely, leptin levels are associated with increased BMD at these sites. However, extensive heterogeneity with regards to sample size, characteristics of study subjects, ethnicity, as well as direction and magnitude of effect at specific skeletal anatomical sites was identified. The broad degree of conflicting findings reported in this study can be attributed several factors. These include differences in study design and ascertainment criteria, the analytic challenges of quantifying specific adipokines and their isoforms, pre‐analytical variables (in particular patient preparation) and confounding effects of co‐existing disease.

**Conclusions:**

This review highlights the biological relevance of adipokines in bone metabolism and reinforces the need for longitudinal research to elucidate the causal relationship of adipokines on bone mass.

## Graphical abstract

A literature search identified 57 articles that explored associations between different adipokines and BMD. Conflicting findings can be partly attributed to the detection of adipokine isoforms, different study designs, patient preparation and confounding effects of comorbid disease. This review highlights the need for a greater understanding of the pathophysiological effects of adipocytokines on bone biology.

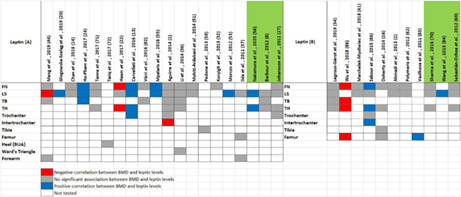

## 1 INTRODUCTION

Adipose tissue is an active endocrine organ and the source of several signalling molecules known as adipokines. It is comprised of a heterogeneous cell population consisting of stromal cells, fibroblasts, macrophages, preadipocytes and adipocytes.^1^ Adipokines have been implicated in the regulation of numerous metabolic processes, including energy metabolism, satiety and interorgan communication.^2^ Dysregulated levels of several adipokines are observed in obesity and type 2 diabetes mellitus (T2DM) and are associated with cardiometabolic traits. This is likely due to the varying effect of different adipokines on chronic inflammation. For example, leptin stimulates the production of various proinflammatory cytokines, whereas adiponectin exerts an inhibitory effect on TNFα‐induced activation of nuclear factor kappa B (NF‐kB).^3^

In addition, adipokines have been variably implicated in osteoporosis. This is a complex progressive metabolic bone disease characterized by microarchitectural deterioration of bone tissue and a reduction in bone mass and strength leading to an increased fracture risk.^4^ In obesity, an increased bone turnover accompanied by a high bone mineral density (BMD) is typically observed. This is an adaptive response to an enlarged body frame potentially modulated by changes in body weight, mechanical loading, differences in bone remodelling, as well as age and gender‐specific changes in body composition.^5^ The role of dysregulated adipokine levels in this process is not clearly understood, and establishing a causal direction remains challenging given the complex and multifactorial nature of the obesity‐BMD interaction.^6^

Receptors for adiponectin, one of the most abundant adipokines in the circulation, are expressed on bone lineage cells.^7^ Obese individuals have reduced circulating levels of adiponectin. This adipokine limits hepatic gluconeogenesis and promotes muscle insulin sensitivity, and a reduction in circulating adiponectin concentrations is reported in obese individuals.^8^

The link between adiponectin and bone physiology is less well understood. Mouse models indicate that adiponectin enhances osteoblastogenesis and promotes bone repair.^9^ In healthy humans, this association holds true. However, the presence of menopause, obesity, metabolic syndrome and other chronic inflammatory states reverses adiponectin's protective effect on bone mass, favouring increased bone resorption.^8^

Leptin mediates the complex crosstalk between the central nervous system (CNS), adipose tissue and energy homeostasis. Its concentration positively correlates with body fat. The expression of leptin receptors on chondrocytes and osteoblasts hints towards its role in bone turnover and endochondral ossification.^10^ *Lep* ^*−/−*^ mice exhibit obesity and reduced femoral neck BMD, bone volume and cortical thickness.^11^ Human studies similarly demonstrate its importance in the maintenance of bone structural integrity, with lower leptin levels linked to abnormal bone microarchitecture and increase fracture risk.^12^ Leptin exhibits angiogenic properties and promotes bone formation via the downregulation of receptor activator of nuclear factor kappa‐Β ligand (RANKL).^13^, ^14^ Additionally, leptin activates RANKL's decoy receptor osteoprotegerin (OPG) and fibroblast growth factor 23 (FGF23), further limiting bone resorption.^15^, ^16^

Gender alters the association between leptin and BMD, with some studies finding non site‐specific correlations between leptin and BMD in females (regardless of menopausal status) but not in males, which can be attributed to the differences in leptin production and leptin sensitivity in males and females.^17^

Resistin is another adipokine that regulates adipogenesis and multiple inflammatory and metabolic processes, although its mechanism of action is not yet fully understood.^18^ Resistin increases the expression of proinflammatory cytokines such as IL‐1, IL‐6 and TNFα, leading to an increased production of reactive oxygen species. Although these effects link resistin to cardiovascular dysfunction,^19^ few studies have been carried out with regards to the association of resistin with bone health, likely due to the relative novelty of this adipokine.

In summary, adipokines are important modulators of bone mass and may demonstrate potential use as bone‐related disease biomarkers. This review aggregates and evaluates evidence from individual studies investigating the effect of adipokines on BMD.

## 2 MATERIALS AND METHODS

A literature search was conducted in the PubMed database to identify studies that examine the relationship between adipokines and BMD. The MeSH search terms ‘adipokines’ and ‘osteoporosis’/‘bone mineral density’/‘bone mass’ were used to select studies, restricting those that have been published between January 2011 and December 2021. Hand‐searching and citation review of relevant studies were also conducted to identify studies not captured by electronic database search. The search terms identified 430 studies, 76 of which were relevant to the review topic.

We selected epidemiological studies carried out in humans that directly quantified adipokine levels in the setting of bone mass and BMD.

Studies were excluded if:

1. They did not directly assess the relationship between adipokines and BMD,
2. Not written in English,
3. Case reports/reviews/editorial letters/case studies,
4. Included paediatric participants,
5. Participants underwent treatment for bone malignancy or
6. Were *in vivo* or *in vitro* functional studies.

**FIGURE 1:**
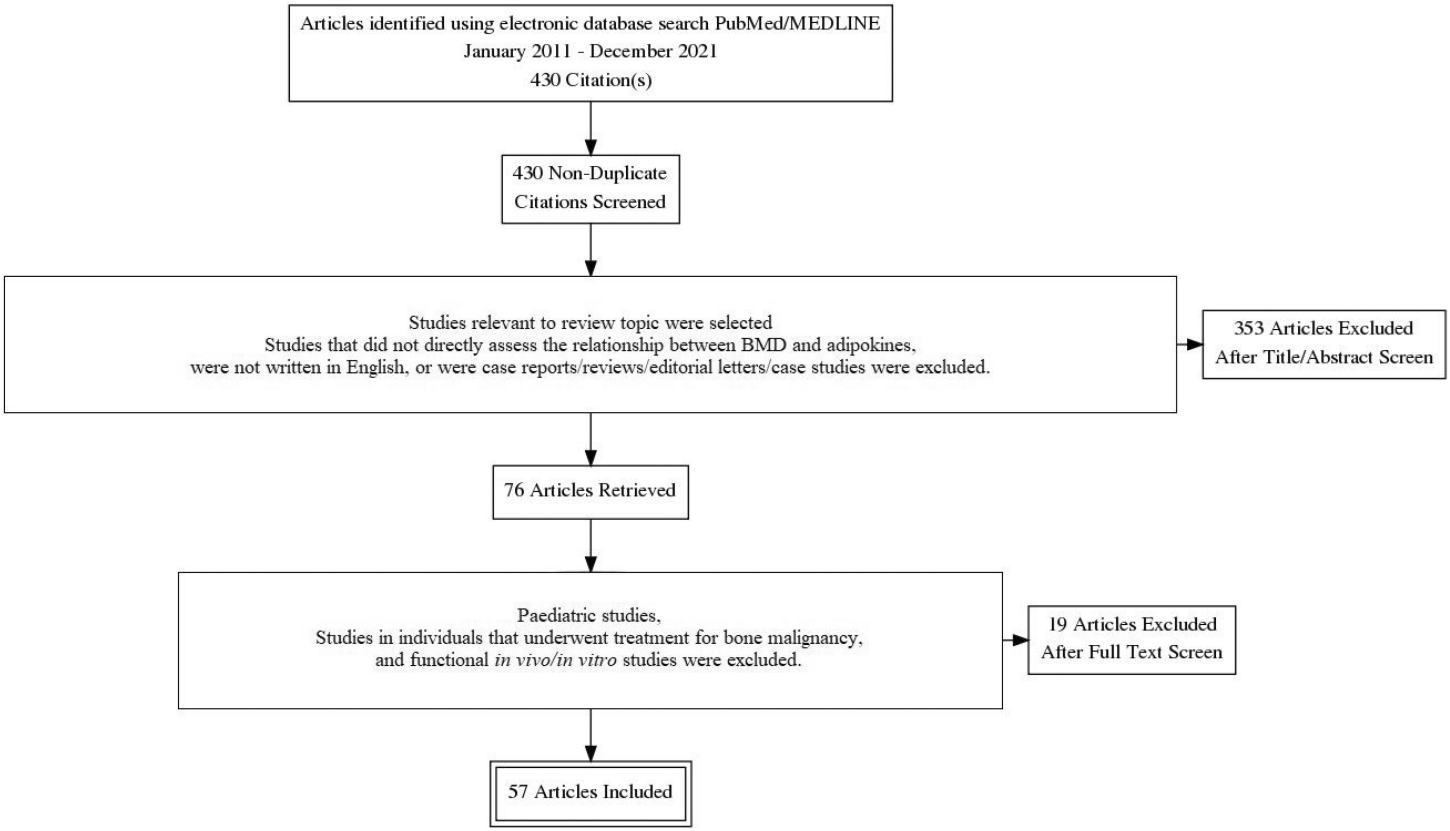
PRISMA flow diagram summarizing the process of selecting articles to be used in this review.

This systematic literature review search is reported in accordance with the Preferred Reporting Items for Systematic Reviews and Meta‐Analyses statement guidelines.^20^ The article selection process is summarized in a Preferred Reporting Items for Systematic Reviews and Meta‐Analyses (PRISMA) flow diagram in Figure 1. Following the review of the article titles and abstracts, study selection and data extraction according to the inclusion and exclusion criteria outlined above were performed. Any discrepancies were adjudicated and resolved by consensus.

The following information was extracted from the selected articles:

1. Primary author and year of publication,
2. Study population,
3. Adipokines assayed and assay methodology and
4. Study outcomes in relation to BMD at specific sites and statistical measures.

## 3 RESULTS

**FIGURE 2:**
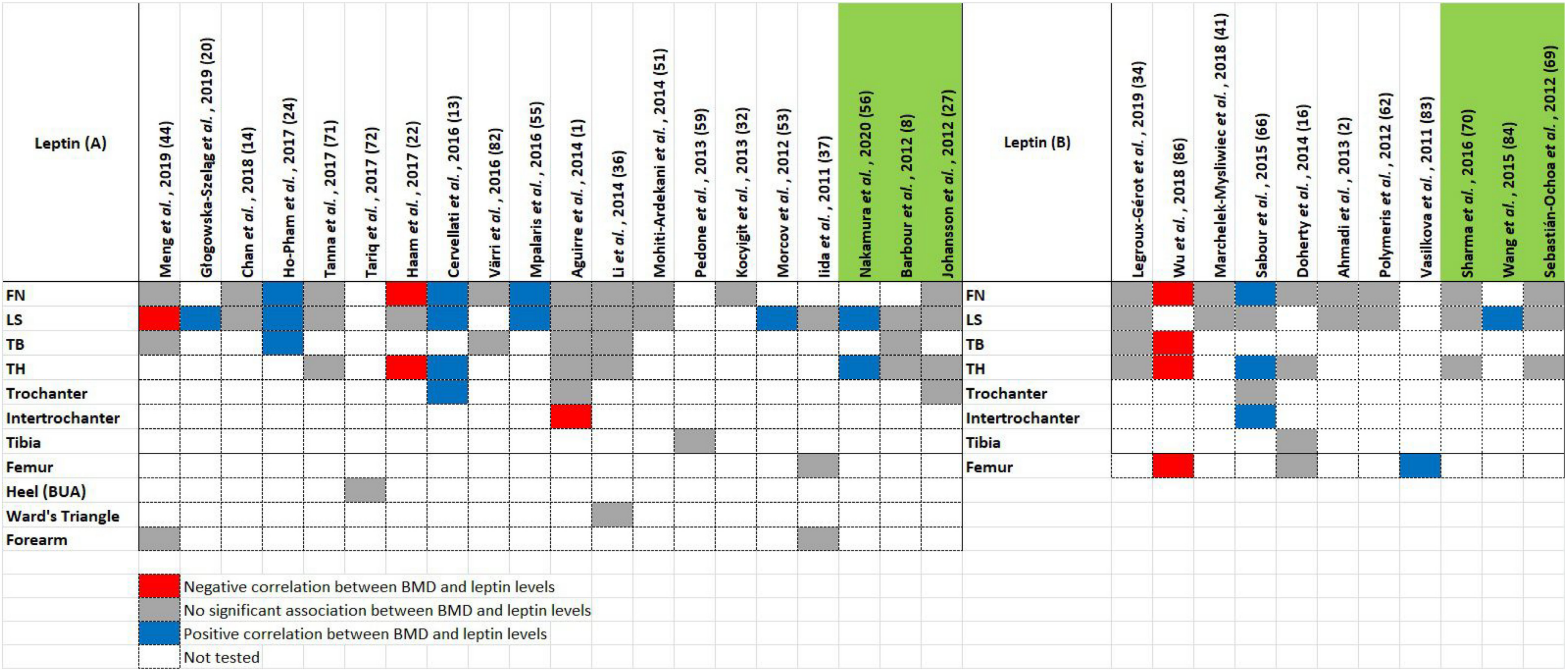
Association of serum leptin levels with BMD at different anatomical sites. (A) Leptin levels in individuals with no comorbidities based on studies described in Table S1; (B) Leptin levels in individuals with comorbidities affecting bone mass based on studies described in Table S2. The first row indicates the study from which the data were derived. The column to the right denotes the site/s of BMD measurement. The studies highlighted in green were longitudinal studies, whilst the rest were case–control studies. Cells highlighted in blue indicate a positive relationship between BMD and leptin. Cells highlighted in red indicate a negative relationship between BMD and leptin. Cells highlighted in grey indicate no significant findings. Cells left blank were not tested. BUA, broadband ultrasound attenuation; FN, femoral neck; LS, lumbar spine; TB, total body; TH, total hip.

**FIGURE 3:**
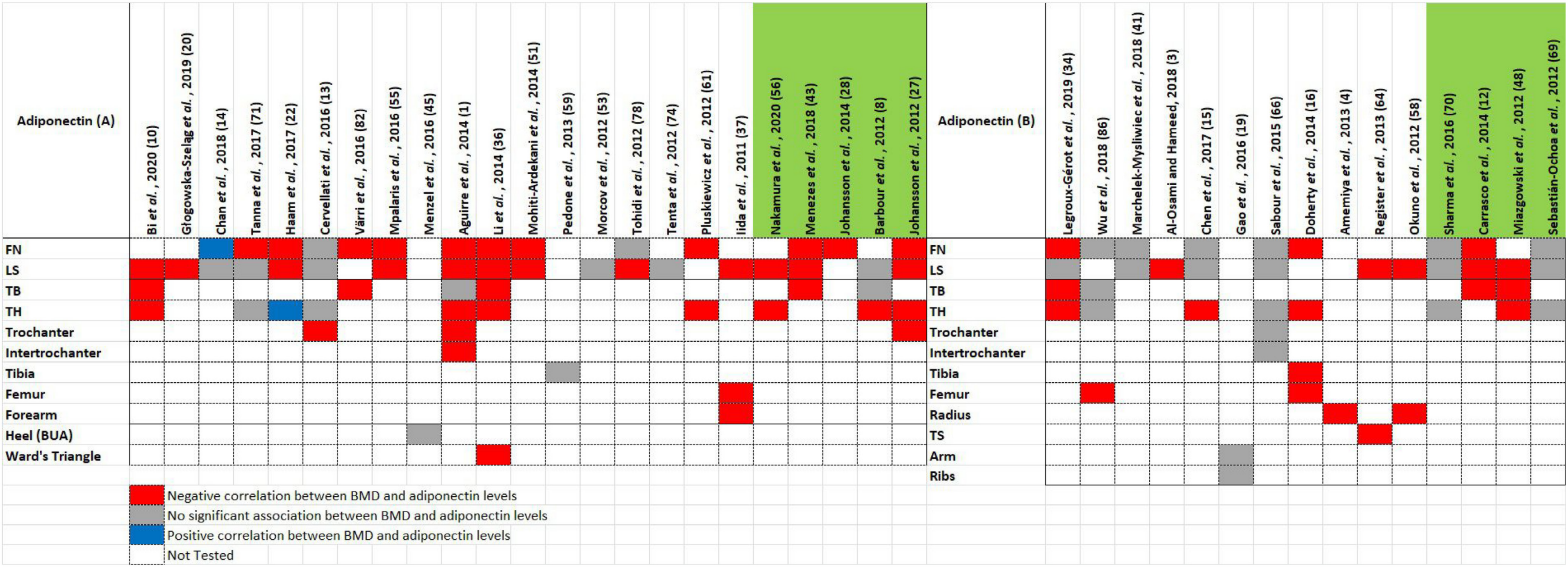
Association of serum adiponectin levels with BMD at different anatomical sites. (A) Adiponectin levels in individuals with no comorbidities based on studies described in Table S1; (B) Adiponectin levels in individuals with comorbidities affecting bone mass based on studies described in Table S2. The first row indicates the study from which the data were derived. The column to the right denotes the site/s of BMD measurement. The studies highlighted in green were longitudinal studies, whilst the rest were case–control studies. Cells highlighted in blue indicate a positive relationship between BMD and adiponectin. Cells highlighted in red indicate a negative relationship between BMD and adiponectin. Cells highlighted in grey indicate no significant findings. Cells left blank were not tested. BUA, broadband ultrasound attenuation; FN, femoral neck; LS, lumbar spine; TB, total body; TH, total hip; TS, thoracic spine.

The literature search yielded 57 studies that assessed the relationship between adipokines and BMD. The studies capture a wide heterogeneity with regards to adipokines, sample size, characteristics of study subjects, ethnicity and direction of effect at specific skeletal sites. Most of the studies were case–control studies focusing on adiponectin and leptin levels in postmenopausal females, utilizing an immunoassay‐based measurement technique. The results of this search have been tabulated in the Tables S1 and S2, with the former table showing studies in participants without comorbidities and the latter highlighting studies with participants diagnosed with comorbidities that may potentially alter BMD or adipokine levels (such as T2DM or spinal cord injury). Unless otherwise stated, BMD was assessed by dual‐energy X‐ray absorptiometry (DXA). The most relevant epidemiological or clinical data from each study have been included. Study findings for leptin and adiponectin are summarized as heat maps in Figures 2 and 3, respectively, stratified by the presence or absence of underlying comorbidities.

Most studies demonstrate that adiponectin and leptin levels exhibit negative and positive correlations, respectively, with BMD. However, other investigations either failed to replicate these findings or reported conflicting associations. With regards to adiponectin, 26 studies reported higher adiponectin levels in participants with a low BMD. Discordant findings were reported by two authors; Chan et al. (2018) identified a positive association between adiponectin levels and FN BMD in elderly Chinese and Indian males of more than 60 years of age, whereas Haam et al. (2017) reported both positive and negative associations depending on the menopausal status of the population studied.^21^, ^22^ Ten studies reported no significant association between BMD and adiponectin.

Of the 31 studies that assessed the association of leptin levels with BMD, four studies reported a negative effect on BMD, nine studies noted a positive association and 18 studies determined no significant association between leptin and BMD. The studies assessed in this review included participants of different ethnicities, age, gender and menopausal status. As a result, establishing a cause–effect relationship between adipokines and BMD remains a considerable challenge. Most of the studies used an enzyme‐linked immunosorbent assay to assess adipokine levels in plasma after an overnight fast. BMD measurements were carried out at the lumbar spine (LS: 43 studies, 74% of studies), femoral neck (FN: 36 studies, 62% of studies) and total hip (TH: 22 studies, 38% of studies). Three studies assessed broadband ultrasound attenuation (BUA) at the right os calcis in lieu of the routine BMD DXA scans. The BUA assessments have been noted to produce comparable results to DXA and therefore have been included in this review.^23^

## 4 DISCUSSION

This article provides an overview of evidence from studies primarily investigating adiponectin and leptin levels in relation to site‐specific BMD. Findings from these studies are characterized by heterogeneity in the direction and magnitude of the relationship between the adipokines and BMD.

Numerous confounding variables may affect the observations presented by the studies outlined in this review, particularly the study designs implemented, leading to discordant results. Meng et al.^24^ assessed the effects of leptin levels on BMD using a Mendelian randomization approach, with genetic variants that lead to increased leptin levels resulting in a reduced LS BMD. The same study did not identify consistent associations at different anatomical sites, which may be a contributing factor to the heterogeneity between studies. Only 11 of the studies analysed in this review were longitudinal cohort studies that assessed the effect of adipokines over an extended period of time, with the shortest study taking place over a 16‐week period^25^ and the longest over 22 years.^26^ The paucity of longitudinal cohort studies thus limits the direct evaluation of the cause–effect relationship between specific adipokines and the incidence of osteoporosis. Cross‐sectional and case–control studies are not designed to evaluate the developmental trajectory of disease and are prone to selection bias, which could partly explain the disparate findings. A longitudinal study carried out by Johansson et al. (2014) reported an attenuated effect of adiponectin over 7.4 years, potentially complicating the assessment of adiponectin levels in cross‐sectional studies. Longitudinal studies also quantified adipokines several years following baseline, potentially missing larger fluctuations during this period.^27^ Scheer et al.^28^ demonstrated that adiponectin levels exhibited circadian fluctuations, with a variation of approximately 40% throughout the day. Disrupted circadian fluctuations can be a potential cause for altered bone formation, and these fluctuations might not be captured by the implemented study designs. Both leptin and adiponectin concentrations are higher in older individuals, possibly due to an increase in fat mass and reduced physical mobility, respectively.^29^ This underscores the need for the development of an age‐specific reference range to be incorporated in future study designs.

Differences in the study population's clinical characteristics contribute to the inconsistent observations. Haam et al. (2017) highlighted these contrasting results by comparing the effect of adiponectin and leptin when stratified by menopausal status and level of obesity in Korean females. Leptin only appeared to negatively influence BMD in the obese postmenopausal females, whereas HMW adiponectin appeared to positively affect BMD in premenopausal females and decrease BMD in postmenopausal females.^22^

Khan et al. (2012) showed that even after adjustment for fat mass, adipokine concentrations differ significantly between different ethnicities in females. African American, Chinese and Japanese females exhibited lower HMW and total adiponectin concentrations and higher leptin concentrations when compared to Caucasian females.^30^ Additionally, genetic variation in *ADIPOQ* and *LEP* genes has been shown to alter adipokine production and function.^31^ Importantly, the risk and progression of osteoporosis as well as the chronic subclinical inflammatory responses characteristic of metabolic diseases are strongly modulated by complex gene–environment interactions that are challenging to quantify. Various drugs such as metformin and statins also modulate inflammatory responses.^32^, ^33^

The direct assessment of adipokine levels in biologically relevant tissue, such as bone matrix, is a potentially superior alternative method to their determination in blood, which overcomes the limitations imposed by improper patient preparation and differences in circulating adipokine half‐life concentrations. Notwithstanding, bone matrix is less accessible to sampling and not ideal for biomarker studies, particularly as it requires extensive processing due to the biological complexity of the tissue.^34^ Some of the study populations investigated in this work were also diagnosed with different comorbidities or underwent interventions (Table S2) which likely affected bone formation and their interactions with adipokines. One of the most commonly encountered comorbidities in the study populations described is T2DM, which has been shown to occasionally complicate DXA measurements on account of the elevated BMD with possible derangement of bone microarchitecture, in addition to the hindrance of bone homeostasis due to an increase in reactive oxygen species.^35^, ^36^ T2DM has also been shown to affect the levels of numerous adipokines.^37^ Spinal cord injury, another common comorbidity in this review, also leads to a drastic reduction in BMD as a result of the loss of mobility.^38^ Adipokines may also have more than one isoform (such as low molecular weight adiponectin and HMW adiponectin). These isoforms may have a variable effect on BMD, possibly due to their altered interaction with oestrogen receptors or other complex mechanisms, especially in the context of ageing, menopause or obesity. More functional studies may aid in elucidating their effects to further understand both the mechanisms of bone loss, establish clinical diagnostic biomarkers, as well as potentially developing novel therapeutic solutions to overcome bone loss in such populations.

The technique used to measure adipokines was not uniform across all of the investigated studies. Many opted to use an enzyme‐based immunoassay, whilst others utilized a radioimmunoassay to measure the analyte in question. Studies have shown that different immunoassays can produce inconsistent results. Diagnostic or research‐based immunoassays can also affect the results obtained. This can provide a potential cause for the incongruent results observed in the studies described above.^39^

Whilst no significant changes in adiponectin concentration can be attributed to the fasting status of the participant,^40^ studies have shown that the same cannot be said for leptin, being significantly reduced in fasted individuals (intermittent and alternate day fasting) as well as those on energy‐restricted diets.^41^ Most of the reviewed studies are based on the analysis of fasting individuals; nonetheless others included non‐fasting individuals which may have inadvertently contributed to the discrepant observations. Other preanalytical variables that may significantly alter the measurement of adipokine levels include the effects of medication on the assessed adipokines, as well as the effects of long‐term storage and freezing. In some of the reviewed studies, the study population was too small to be able to reach the necessary statistical power to ascertain the observations noted. This is especially evident in studies listed in Table S2. Weaker associations between adipokines and BMD may remain unnoticed through the use of smaller populations.^42^ This review shows that a causal direction between adipokines and BMD cannot be robustly inferred. Large biobank‐driven longitudinal cohort studies could facilitate a better understanding of the role of these adipokines with regards to bone health.

## 5 CONCLUSION

Whilst a net positive and negative association can be observed between BMD and leptin or adiponectin, respectively, various factors appear to contribute to the discordant findings demonstrated in this review. The differences in study population characteristics, such as gender and menopausal status, appear to be the most evident contributors to the inconsistency of the results. The lack of longitudinal studies also questions the causal direction between adipokines and an altered bone mass. Further research into adipokines other than leptin and adiponectin is also warranted to potentially identify new associations with BMD, possibly paving the way for novel diagnostic biomarkers.

## AUTHOR CONTRIBUTIONS

**Darren Mangion:** Data curation (equal); writing – original draft (equal). **Nikolai P. Pace:** Conceptualization (equal); funding acquisition (equal); project administration (equal); supervision (equal); writing – review and editing (equal). **Melissa M. Formosa:** Conceptualization (equal); funding acquisition (equal); project administration (equal); supervision (equal); writing – review and editing (equal).

## CONFLICT OF INTEREST

All authors declare no conflict of interest.

## ETHICAL APPROVAL

Ethics approval was not required since this is a systematic review of previously published studies.

## Supporting information


Appendix S1.

## Data Availability

The data that support the findings of this study are available in the public domain MEDLINE searchable via PubMed at https://pubmed.ncbi.nlm.nih.gov/.
