# Supplementary material for: The relationship between adipokine levels and bone mass—A systematic review
Source: Endocrinol Diabetes Metab. 2023 Feb 9;6(3):e408. doi: 10.1002/edm2.408 (PMC10164433; doi:10.1002/edm2.408)
Supplement: Supplementary file 1 — Appendix S1. [file EDM2-6-e408-s001.docx]

**Supplementary Tables**

**Supplementary Table 1**. **Relationship of Adipokine levels with BMD in participants without comorbidities.** The table summarises the results of 34 studies detailing the adipokines assessed, the study population’s demographics and any associations noted with BMD. Studies below the line were longitudinal. If not stated, adipokine measurement was carried out using an immunoassay-based technique, or the information was unavailable. Unless otherwise stated, BMD measurement was performed by DXA.

| **Study Reference** | **Year** | **Population** | **Adipokines Studied** | **BMD Findings** | | | | | | | | | | | | |
| --- | --- | --- | --- | --- | --- | --- | --- | --- | --- | --- | --- | --- | --- | --- | --- | --- |
| Tariq *et al.*, 2021 ^1^ | 2021 | 160 post-menopausal females (70 non-osteoporotic with a mean age of 54 years [50 - 57], 90 osteoporotic with a mean age of 62 years [55 – 67]) | Resistin | LS (L2-L4)  ↓ | | | | Right FN  ↓ | | Right Hip  ↓ | | | | Left FN  ↓ | | Left Hip  ↓ |
| Bi *et al.*, 2020 ^2^ | 2020 | 112 males (mean age of 43.6 ± 14.1) and 188 females (mean age of 43.2 ± 13.5) from Singapore | Adiponectin (males) | TB  NS | | | Hip  NS | | | | | | | LS  NS | | |
|  |  |  | Adiponectin (females) | ↓ | | | ↓ | | | | | | | ↓ | | |
| Meng *et al.*, 2019 ^3^ | 2019 | 52,140 participants of European ancestry from the Genetic factors for osteoporosis (GEFOS) consortium | Leptin | FN  NS | | | | LS  ↓ | Forearm  NS | | | | | TB  NS | | |
|  |  |  | *LEP* rs10487505, *GCKR* rs780093, *CCNL1* rs900400, *SLC32A1* rs6071166, and *COBLL1* rs6738627 were known to be positively associated with leptin concentration, and so were used for this Mendelian randomization study | | | | | | | | | | | | | |
| Mihai *et al.*, 2019 ^4^ | 2019 | 61 females diagnosed with primary osteoporosis, with females with a normal BMI having a mean age of 65.69 ± 9.66 years and overweight/obese females having a mean age of 67.58 ± 5.80 years | Visfatin | LS (L1-L4)  NS | | | | | | FN  NS | | | | | | |
|  |  |  | Retinol-binding protein 4 (RBP-4) | ↑ | | | | | | NS | | | | | | |
| Głogowska-Szeląg *et al.*, 2019 ^5^ | 2019 | 80 post-menopausal females diagnosed with osteoporosis with varying BMI | Leptin | LS  ↑ | | | | | | | | | | | | |
|  |  |  | Adiponectin  Resistin | NS  NS | | | | | | | | | | | | |
| Xu *et al.*, 2018 ^6^ | 2018 | 200 post-menopausal females diagnosed with osteoporosis (mean age of 67.5 ± 7.4 years) matched with 187 healthy control females (mean age of 67.8 ± 6.9 years) | C1q/TNF-Related Protein 3 (CTRP3) | LS (L2-L4)  ↑ | | | | | FN  ↑ | | | | | | | |
|  |  |  | The novel adipokine CTRP3 was decreased in females diagnosed with osteoporosis when compared with controls | | | | | | | | | | | | | |
| Menzel *et al.*, 2018 ^7^ | 2018 | 683 females, 404 of which were pre/peri-menopausal (mean age of 41.3 ± 4.5 years) and 279 of which were post-menopausal (mean age of 59.4 ± 3.6 years) | Chemerin (pre/peri-menopausal) | Right os calcis (broadband ultrasound attenuation (BUA))  ↓ | | | | | | | | | | | | |
|  |  |  | Chemerin (post-menopausal) | NS | | | | | | | | | | | | |
| Kadric *et al.*, 2018 ^8^ | 2018 | 3583 adults (1780 males, 1803 females) of varying BMI. The study population was aged 20 to 79 years | Chemerin | Right os calcis (BUA)  ↓ | | | | | | | | | | | | |
|  |  |  | This study only noted a negative correlation between chemerin and BUA of the right os calcis in obese males and females. The effect of this adipokine appears dependent on the participant’s BMI. | | | | | | | | | | | | | |
| Chan *et al.*, 2018 ^9^ | 2018 | 120 males (60 Indian and 60 Chinese) aged 60 years or older with a mean age of 66.24 ± 5.97 years | Leptin  Resistin | LS  NS  NS | | | | | | FN  NS  NS | | | | | | |
|  |  |  | Adiponectin | NS | | | | | | ↑ | | | | | | |
| Ho-Pham *et al.*, 2017 ^10^ | 2017 | 611 individuals (413 females aged 47.5 ± 17.0 years and 198 males aged 43.6 ± 18.0) | Leptin (Males) | FN  ↑ | | | LS (L2-L4)  ↑ | | | | | | | TB  ↑ | | |
|  |  |  | Leptin (Females) | ↑ | | | ↑ | | | | | | | NS | | |
|  |  |  | The variance attributed to leptin levels was 0.5%. Results were all not significant when the effects of fat mass were removed. Observations were unadjusted for fat mass. | | | | | | | | | | | | | |
| Moradi *et al.*, 2017 ^11^ | 2017 | 312 obese Iranian females with a mean age of 39.85 ± 12.06 years | RBP-4  Angiopoietin-like 6 (ANGPL6)  Vaspin | Total BMD at Femur  NS  NS  NS | | | | | | LS (L2-L4)  NS  NS  NS | | | | | | |
|  |  |  | Omentin-1 | ↑ | | | | | | NS | | | | | | |
|  |  |  | The positive effect of omentin-1 was noted to be due to the influence on the resting metabolic rate omentin-1 has in these individuals. | | | | | | | | | | | | | |
| Tanna *et al.*, 2017 ^12^ | 2017 | 386 ambulant post-menopausal females with a mean age of 61 ± 6.4 years | Adiponectin | LS  NS | | | FN  ↓ | | | | | | | TH  NS | | |
|  |  |  | Vaspin | NS | | | ↑ | | | | | | | ↑ | | |
|  |  |  | Leptin | NS | | | NS | | | | | | | NS | | |
|  |  |  | Adiponectin was found in increased quantities in females who sustained fractures and was linked to an increased incidence of fractures. | | | | | | | | | | | | | |
| Tariq *et al.*, 2017 ^13^ | 2017 | 192 Pakistani females consisting of 98 post-menopausal females (58.42 ± 3.56 years) and 94 pre-menopausal females (mean age of 57.92 ± 4.31 years) | Leptin | Heel (by quantitative ultrasound)  NS | | | | | | | | | | | | |
| Haam *et al.*, 2017 ^14^ | 2017 | 255 Korean females, 158 of which were pre-menopausal (mean age of 41 [38.0–46.0] years) and 97 were post-menopausal (mean age of 55 [51.0–59.0] years) | Adiponectin | LS (L1-L4)  NS | | | TH  ↑‡ | | | | | | | FN  ↓† | | |
|  |  |  | HMW adiponectin | ↓† | | | ↑‡ | | | | | | | ↓† | | |
|  |  |  | Leptin | NS | | | ↓† | | | | | | | ↓† | | |
|  |  |  | This study demonstrated adipokine interactions with BMD in females with and without central obesity.  † Post-menopausal females without central obesity.  ‡ Pre-menopausal females without central obesity. | | | | | | | | | | | | | |
| Cervellati *et al.*, 2016 ^15^ | 2016 | 127 females in post-menopausal status (mean age of females with a normal BMD – 55.3 ± 0.7, Osteopenic – 56.7 ± 0.6, Osteoporotic – 58.5 ± 0.4) | Leptin | LS  ↑ | | | FN  ↑ | | | Trochanter  ↑ | | | | TH  ↑ | | |
|  |  |  | Adiponectin | NS | | | NS | | | ↓ | | | | NS | | |
|  |  |  | Resistin  Hepatocyte Growth Factor Adipsin | NS  NS  NS | | | NS  NS  NS | | | NS  NS  NS | | | | NS  NS  NS | | |
|  |  |  | IL-6 may influence the effect of adiponectin on bone, with lower levels of IL-6 leading to an increased inverse association between adiponectin and bone. | | | | | | | | | | | | | |
| Värri *et al.*, 2016 ^16^ | 2016 | 290 post-menopausal females with a mean age of 73.6 ± 2.8 years | HMW Adiponectin | FN  ↓ | | | | | | TB  ↓ | | | | | | |
|  |  |  | Leptin | NS | | | | | | NS | | | | | | |
| Mpalaris *et al.*, 2016 ^17^ | 2016 | 110 post-menopausal females with a mean age of 60 (55–68) years | Leptin | LS (L2-L4)  ↑ | | | | | | FN  ↑ | | | | | | |
|  |  |  | Adiponectin | ↓ | | | | | | ↓ | | | | | | |
|  |  |  | Ghrelin | NS | | | | | | NS | | | | | | |
| Menzel *et al.*, 2016 ^18^ | 2016 | 637 females, 388 of which were peri-/premenopausal (mean age of 41.2 ± 4.6 years) whilst 249 were post-menopausal (mean age of 59.0 ± 3.6 years) | Omentin-1 (pre-menopausal) | Right os calcis (BUA)  NS | | | | | | | | | | | | |
|  |  |  | Omentin-1 (post-menopausal) | ↓ | | | | | | | | | | | | |
|  |  |  | Adiponectin | NS | | | | | | | | | | | | |
|  |  |  | This study utilized BUA for measurement of bone mineral density of the right os calcis, which correlates strongly with BMD | | | | | | | | | | | | | |
| Zheng *et al.*, 2015 ^19^ | 2015 | 744 post-menopausal females with mean ages ranging from 58.0 ± 7.7 years in the first quartile to 61.0 ± 8.1 years in the fourth quartile (by DPP4 concentration) | Dipeptidyl peptidase-4 (DPP4) | FN  ↓ | | | | | LS (L1-L4)  ↓ | | | | | | | |
|  |  |  | This study noted that higher concentrations of DPP4 were associated with lower BMD values at the femoral neck and lumbar spine (at a p-value of less than 0.05). Participants in the highest quartile of DPP4 had a significantly higher risk of developing osteoporosis. | | | | | | | | | | | | | |
| Aguirre *et al.*, 2014 ^20^ | 2014 | 173 elderly, frail individuals (81 males, 92 females) with a mean age of 69.5 ± 4.2 years | Adiponectin (Females) | TB  NS | LS  ↓ | | | TH  ↓ | | FN  NS | | | Tro  NS | | Intertro  ↓ | |
|  |  |  | Adiponectin (Males) | NS | NS | | | NS | | ↓ | | | ↓ | | ↓ | |
|  |  |  | Leptin | NS | NS | | | NS | | NS | | | NS | | ↓† | |
|  |  |  | IL-6, high-sensitivity C-reactive protein (hs-CRP) | NS | NS | | | NS | | NS | | | NS | | NS | |
|  |  |  | This study indicated that in females, adiponectin appears to modulate the negative association observed between fat mass and BMD.  †Association only noted in males. | | | | | | | | | | | | | |
| Li *et al.*, 2014 ^21^ | 2014 | 219 healthy Chinese males with a mean age of 46.37 ± 15.67 years | Adiponectin | LS (L1-L4)  ↓ | | | TH  ↓ | | | | FN  ↓ | | | Ward’s  ↓ | | TB  ↓ |
|  |  |  | Omentin – 1  Leptin  Resistin | NS  NS  NS | | | NS  NS  NS | | | | | NS  NS  NS | | NS  NS  NS | | NS  NS  NS |
| Mohiti-Ardekani *et al.*, 2014 ^22^ | 2014 | 81 non-diabetic individuals diagnosed with osteoporosis (with a mean age of 54.5 ± 15.5 years) with 120 controls (with a mean age of 39.7 ± 10.4 years) | Adiponectin | LS (L1-L4)  ↓† | | | | | FN  ↓† | | | | | | | |
|  |  |  | Leptin | NS | | | | | NS | | | | | | | |
|  |  |  | Resistin | NS | | | | | ↓† | | | | | | | |
|  |  |  | †These results were only noted in the osteoporosis group. | | | | | | | | | | | | | |
| Pedone *et al.*, 2013 ^23^ | 2013 | 690 elderly patients (397 females, 293 males) with a mean age of 75.2 ± 7.6 years | Leptin  Adiponectin  Resistin | Tibia  NS  NS  NS | | | | | | | | | | | | |
| Kocyigit *et al.*, 2013 ^24^ | 2013 | 42 post-menopausal females (mean age of 58.2 ± 6.4 years) diagnosed with osteoporosis matched with 37 post-menopausal healthy control females (mean age of 59.2 ± 7.8 years) | Leptin | FN  NS | | | | | | | | | | | | |
| Morcov *et al.*, 2012 ^25^ | 2012 | Six groups of 8-15 pre-menopausal and post-menopausal females, with varying BMI and an age distribution of 31 and 37 respectively | Adiponectin | LS  NS | | | | | | | | | | | | |
|  |  |  | Leptin | ↑ | | | | | | | | | | | | |
| Tohidi *et al.*, 2012 ^26^ | 2012 | 382 post-menopausal Iranian females with a mean age of 59.0 ± 7.5 years | Omentin-1 | LS (L2-L4)  ↓ | | | | | Proximal FN  NS | | | | | | | |
|  |  |  | Visfatin | NS | | | | | NS | | | | | | | |
|  |  |  | Adiponectin | ↓† | | | | | NS | | | | | | | |
|  |  |  | †When this study compared the highest quartile of adiponectin to that of the lowest quartile, a negative effect on BMD at the lumbar spine was revealed. | | | | | | | | | | | | | |
| Tenta *et al.*, 2012 ^27^ | 2012 | 81 post-menopausal Greek females, 38 of which were healthy (mean age of 53.7 ± 4.5 years) and 43 of which were osteopenic/osteoporotic (mean age of 55.5 ± 4.2 years) | Adiponectin (Total & HMW) | LS (L2-L4)  NS | | | | | | | | | | | | |
| Pluskiewicz *et al.*, 2012 ^28^ | 2012 | 80 females from 625 participants (40 of which had the lowest BMD and 40 of which had the highest BMD after stratifying into six age groups and taking the lowest and highest BMD) with a mean age of 66.1 ± 8.0 years | Adiponectin | FN  ↓† | | | | | TH  ↓ | | | | | | | |
|  |  |  | Resistin | NS | | | | | NS | | | | | | | |
|  |  |  | A significant difference was noted in adiponectin concentrations between the two groups.  †After stepwise multiple regression analysis, only total hip BMD was negatively correlated with adiponectin. | | | | | | | | | | | | | |
| Iida *et al.*, 2011 ^29^ | 2011 | 111 female participants (aged 40 to 80 years) | Adiponectin (post-menopausal) | LS (L2-L4)  NS | | | Femoral  NS | | | | | | | Distal Forearm  ↓ | | |
|  |  |  | Adiponectin (Pre-menopausal) | ↓ | | | ↓ | | | | | | | NS | | |
|  |  |  | Leptin | NS | | | NS | | | | | | | NS | | |
| Nakamura *et al.*, 2020 ^30^ | 2020 | 1167 post-menopausal Japanese females with a mean age of 65.9 years | Leptin | LS  ↑ | | | | | Hip  ↑ | | | | | | | |
|  |  |  | Adiponectin | ↓ | | | | | ↓ | | | | | | | |
|  |  |  | This was a 72-year follow-up study. This study concluded that a raised adiponectin lead to an increase risk of vertebral fractures whereas decreased concentrations of leptin increased the risk of fractures at the long-bone. | | | | | | | | | | | | | |
| Menezes *et al.*, 2018 ^31^ | 2018 | 3523 live births in Brazil (1663 males and 1860 females) with analysed IL-6 and CRP. A random sample of 1706 from the above included adiponectin measurement | Adiponectin | TB  ↓‡ | | | LS  ↓‡,† | | | | | | | FN  ↓‡ | | |
|  |  |  | Live births in 1993 were followed up in Brazil at ages 11, 15, 18 and 22 years of age. Samples were collected at ages 18 and 22 years for measurement of adiponectin. BMD was measured at 22 years  † Associations noted in females  ‡ Associations noted in males  CRP was analysed by an immunoturbidimetric assay | | | | | | | | | | | | | |
| Johansson *et al.*, 2014 ^32^ | 2014 | 989 elderly males with a mean age of 75.3 ± 3.2 years, 124 of which sustained fractures, with a mean age of 75.5 ± 3.2 years | Adiponectin | FN  ↓ | | | | | | | | | | | | |
|  |  |  | This was a longitudinal study which followed up the subjects for up to 7.4 years. Samples for quantification of adipokines and BMD were assessed at recruitment.  This study noted that whilst there is an association between increased adiponectin levels and fracture risk, adiponectin may be unsuitable for use in long-term risk management as the effect is attenuated by time. | | | | | | | | | | | | | |
| Barbour *et al.*, 2012 ^33^ | 2012 | 3,075 participants living in Pittsburgh and Memphis; 1584 were females (mean age of 73.5 ± 2.9) and 1491 were males (mean age of 73.8 ± 2.9 | Adiponectin (males) | TB  NS | | | Hip  NS | | | | | | | LS (L3)  NS | | |
|  |  |  | Adiponectin (females) | NS | | | ↓ | | | | | | | NS | | |
|  |  |  | Leptin | NS | | | NS | | | | | | | NS | | |
|  |  |  | In this study, the participants’ adiponectin and leptin concentrations were obtained at the beginning of the study, alongside the hip and whole body BMD. Trabecular lumbar spine BMD was also calculated. Hip and whole-body BMD measurements were collected on year 3, 5/6, 8 and 10. Trabecular lumbar spine BMD was recollected on year 6. The degree of bone loss throughout the years was correlated with adiponectin and leptin concentrations at baseline. | | | | | | | | | | | | | |
| Johansson *et al.*, 2012 ^34^ | 2012 | 999 elderly males with a mean age of 75.2 ± 3.2 years, 150 of which sustained fractures, with a mean age of 75.5 ± 3.2 years. | Adiponectin | TH  ↓ | | LS  ↓ | | | FN  ↓ | | | | | Trochanteric  ↓ | | |
|  |  |  | Leptin | NS | | NS | | | NS | | | | | NS | | |
|  |  |  | This was a longitudinal study which followed up the subjects for up to 7.4 years. Samples for quantification of adipokines and BMD were assessed at recruitment.  The study noted a positive association between increased adiponectin levels and fractures. | | | | | | | | | | | | | |

↓ denotes an observed negative effect on BMD

↑ denotes an observed positive effect on BMD

FN: Femoral neck, FS: Femoral shaft, Intertro: Intertrochanteric, LS: Lumbar spine, NS: non-significant, TB: Total body, TF: Total femur, TH: Total hip, TS: Thoracic spine.

**Supplementary Table 2.** **Relationship of Adipokine levels with BMD in participants with comorbidities or who underwent interventions.** The table summarises the results of 23 studies detailing the adipokines assessed, the study population’s demographics and any associations noted with BMD. Studies below the line were longitudinal. If not stated, adipokine measurement was carried out using an immunoassay-based technique, or the information was unavailable. Unless otherwise stated, BMD measurement was performed by DXA.

| **Study Reference** | **Year** | **Population** | **Adipokines Studied** | **BMD Findings** | | | | | | | | | | | | | | |
| --- | --- | --- | --- | --- | --- | --- | --- | --- | --- | --- | --- | --- | --- | --- | --- | --- | --- | --- |
| Yan *et al.*, 2020 ^35^ | 2020 | 172 individuals diagnosed with type-2 diabetes; 52 (41 males, 11 females) had a normal BMD and had a mean age of 61.12 ± 8.98, 66 (34 males, 32 females) were osteopenic and had a mean age of 64.45 ± 9.80, 54 (14 males, 40 females) were osteoporotic and had a mean age of 67.78 ± 8.56 | Omentin-1 | LS  ↓ | | | FN  ↓ | | | | | TH  ↓ | | | | | | |
|  |  |  | This study determined that individuals diagnosed with osteoporosis or osteopenia exhibited raised omentin-1 levels. | | | | | | | | | | | | | | | |
| Legroux-Gérot *et al.*, 2019 ^36^ | 2019 | 80 female anorexia nervosa patients with a mean age of 23.8 ± 4.7 years | Leptin | TB  NS | | LS  NS | | | | | FN  NS | TH  NS | | | | | | |
|  |  |  | Total Adiponectin | ↓ | | NS† | | | | | ↓ | ↓ | | | | | | |
|  |  |  | HMW Adiponectin | NS† | | NS† | | | | | ↓‡ | ↓ | | | | | | |
|  |  |  | Preadipocyte factor 1 (Pref-1) | NS | | NS | | | | | NS§ | NS | | | | | | |
|  |  |  | The table above represents the entire study population. When segregated by osteoporosis groups and by BMI of less than 17.5 group:  † Denotes a negative correlation in both osteoporosis group and BMI group compared to the rest of the study population (osteopenic, BMI more than 17kg/m^2^ and controls).  ‡ NS in both osteoporosis group and BMI group.  § Positively correlated in BMI group. | | | | | | | | | | | | | | | |
| Wu *et al.*, 2018 ^37^ | 2018 | 164 patients (145 females, 19 males) with knee osteoarthritis (with a mean age of 55.4 years) | Leptin | TB  ↓ | | | | Hip  ↓ | | TF  ↓ | | FN  ↓ | | | | | FS  ↓ | |
|  |  |  | Adiponectin | NS | | | | NS | | ↓ | | NS | | | | | ↓ | |
|  |  |  | Resistin | NS | | | | NS | | NS | | NS | | | | | NS | |
| Huang *et al.*, 2018 ^38^ | 2018 | 274 Han Chinese older than 50 years of age (with a mean age of 63.26 ± 8.16 years) diagnosed with T2DM, 148 of which had normal BMD, 93 were osteopenic and 33 were osteoporotic | RBP-4 | FN  ↑ | | | | | TH  ↑ | | | | | | | LS (L1-L4) | | |
|  |  |  | RBP-4 measurement was carried out by turbidimetric inhibition immunoassay | | | | | | | | | | | | | | | |
| Marchelek-Mysliwiec *et al.*, 2018 ^39^ | 2018 | 52 patients with chronic kidney disease with a mean age of 60.07 ± 9.8 years and 23 control individuals with a mean age of 53.7 ± 13.2 years. | Leptin  Adiponectin | FN  NS  NS | | | | | | | | LS (L1-L4)  NS  NS | | | | | | |
| Al-Osami & Hameed, 2018 ^40^ | 2018 | 90 postmenopausal females (30 type-2 diabetic and osteoporotic, 30 non-diabetic and osteoporotic, 30 healthy). Mean ages ranged from 60.18 ± 6.47 years in the control group to 62.33 ± 8.41 years in group A | Adiponectin | LS (L1-L4)  ↓† | | | | | | | | | | | | | | |
|  |  |  | †The negative correlation between lumbar BMD and adiponectin was noted in osteoporotic females diagnosed with T2DM. | | | | | | | | | | | | | | | |
| Chen *et al.*, 2017 ^41^ | 2017 | 71 males diagnosed with obstructive sleep apnea syndrome (OSAS) and 13 control males. The control group had an average age of 41.33 ± 12.45, Moderate OSAS group had an average age of 43.35 ± 11.95 and severe OSAS group had an average age of 42.06 ± 11.75 | Adiponectin | LS (L1-L4)  NS | | | | | FN  NS | | | | | | TH  ↓ | | | |
| Gao *et al.*, 2016 ^42^ | 2016 | 52 females diagnosed with polycystic ovary syndrome (PCOS) (with a mean age of 22.5 years) and 39 control females (with a mean age of 26 years) | Irisin (PCOS Group) | Right Arm  NS | | | | Right Ribs  NS | | | | TB BMD T-score  ↓ | | | | | TB BMD Z-score  NS | |
|  |  |  | Irisin (Control Group) | ↑ | | | | ↑ | | | | ↑ | | | | | ↑ | |
|  |  |  | Adiponectin | NS | | | | NS | | | | NS | | | | | NS | |
| Terzoudis *et al.*, 2016 ^43^ | 2016 | 120 individuals suffering from inflammatory bowel disease (64 males, 56 females) together with 98 healthy matched controls. Cases had a mean age of 47.41 ± 16.1 years | Chemerin | LS (L1-L4)  ↓ | | | | | | | | FN  ↓ | | | | | | |
|  |  |  | Visfatin | ↓ | | | | | | | | ↓ | | | | | | |
|  |  |  | Vaspin | NS | | | | | | | | NS | | | | | | |
| Sabour *et al.*, 2015 ^44^ | 2015 | 104 patients with a diagnosed spinal cord injury. Participants comprised of 85 males with a mean age of 51.80 ± 13.44 and 19 females with a mean age of 56.05 ± 7.89 | Leptin | LS (L1-L4)  NS | Trochanter  NS | | | | | Intertro  ↑† | | FN  ↑† | | | | | | TH  ↑† |
|  |  |  | Adiponectin | NS | NS | | | | | NS | | NS | | | | | | NS |
|  |  |  | † Observations only noted in female participants, and should be interpreted with caution due to a low sample population | | | | | | | | | | | | | | | |
| Doherty *et al.*, 2014 ^45^ | 2014 | 149 males diagnosed with chronic spinal cord injury, 54 of which were mobile with a mean age of 62.6 ± 12.0 years and 95 of which were wheelchair users with a mean age of 51.3 ± 12.5 years | Adiponectin | Distal femur  ↓ | | Proximal tibia  ↓ | | | | | FN  ↓ | | | TH  ↓ | | | | |
|  |  |  | Leptin | NS | | NS | | | | | NS | | | NS | | | | |
|  |  |  | When further dividing the participants based on walking status, it was revealed that individuals that are wheelchair bound had a negative correlation between adiponectin. The effect was not noted in mobile individuals. | | | | | | | | | | | | | | | |
| Ahmadi *et al.*, 2013 ^46^ | 2013 | 72 patients on haemodialysis (43 males, 29 females) with a mean age of 55.1 ± 11.4 years. | Leptin | FN  NS | | | | | | | | LS  NS | | | | | | |
| Amemiya *et al.*, 2013 ^47^ | 2013 | 70 males (mean age of 65.8 ± 8.9) and 22 females (mean age of 68.9 ± 10.4) on haemodialysis. | Adiponectin (HMW) | 1/3 Distal Radius  ↓ | | | | | | | | | | | | | | |
| Register *et al.*, 2013 ^48^ | 2013 | 479 African American participants (272 females and 207 males) with type-2 diabetes and a mean age of 55.6 ± 9.5 years. | Adiponectin | TS (T8-T11)  ↓ | | | | | | | LS (T12-L3)  ↓ | | | | | | | |
| Okuno *et al.*, 2012 ^49^ | 2012 | 114 male patients aged between 49.9 – 72.1 years who have been on hemodialysis for a period ranging from 3.6 – 9.6 years | Adiponectin | 1/3 Distal Radius  ↓ | | | | | Ultra-distal Radius  ↓ | | | LS (L2-L4)  ↓ | | | | | | |
| Polymeris *et al.*, 2012 ^50^ | 2012 | 37 patients on haemodialysis (18 females, with a mean age of 56.7 years and 19 males with a mean age of 58 years) | Leptin | LS  NS | | | | | | | | FN  NS | | | | | | |
|  |  |  | This study concluded that the reduced bone mass prevalent in haemodialysis patients can be attributed to the increased circulating parathyroid hormone, and is not correlated with 25OH Vitamin D or leptin. | | | | | | | | | | | | | | | |
| Vasilkova *et al.*, 2011 ^51^ | 2011 | 168 Belarusian males diagnosed with T2DM (mean age of 54.1 ± 4.8 years). | Leptin | Left Femur  ↑ | | | | | | | | | | | | | | |
| Bassatne *et al.*, 2020 ^52^ | 2020 | 257 elderly patients (129 assigned high dose vitamin D3, 128 low dose vitamin D3) with a mean age of 71.1 ± 4.7 years | DLK1 | Subtotal  NS | | Total LS  NS | | | | | TH  NS | | | FN  ↓ | | | | |
|  |  |  | DLK1 and subtotal, total lumbar spine, total hip and femoral neck BMD was analysed at baseline and after 12 months in this longitudinal study | | | | | | | | | | | | | | | |
| Sharma *et al.*, 2016 ^53^ | 2016 | 318 females diagnosed with HIV (Mean age of 43.1 years) and 122 uninfected female controls (Mean age of 37 years). | Adiponectin  Leptin | LS  NS  NS | | | | | TH  NS  NS | | | FN  NS  NS | | | | | | |
|  |  |  | This study measured the lumbar spine, total hip and femoral neck BMD of the participants over a period of five years alongside adipokine levels to assess their effect (alongside HIV status) on BMD. | | | | | | | | | | | | | | | |
| Wang *et al.*, 2015 ^54^ | 2015 | 15 male patients suffering from spinal cord injury with a mean age of 46.60 ± 11.88 years of age, with a fracture at lower extremities. Patients matched with controls with a mean age of 44.47 ± 12.09 years of age without spinal cord injury suffering from similar fractures at lower extremities. | Leptin | LS (L1-L4)  ↑† | | | | | | | | | | | | | | |
|  |  |  | †Serum leptin positively correlated with LS BMD in the control group only.  Serum leptin and BMD was noted at 2, 4, 8 and 16 weeks from fracture treatment.  No rise in leptin levels were noted in the control group, whilst higher leptin levels were noted in the individuals suffering from spinal cord injuries. A rise in BMD was noted in the control group after 2, 4 and 8 weeks. | | | | | | | | | | | | | | | |
| Carrasco *et al.*, 2014 ^55^ | 2014 | 43 non-menopausal females (23 undergoing gastric bypass with a mean age of 37.3 ± 8.1 and 20 undergoing sleeve gastrectomy with a mean age of 34.2 ± 10.2) | Total ghrelin | TB  ↑ | | | | | LS (L2-L4)  ↑ | | | | FN  ↑ | | | | | |
|  |  |  | Adiponectin | ↓† | | | | | ↓† | | | | ↓† | | | | | |
|  |  |  | This study collected measurements on BMD, ghrelin and adiponectin before and 12 months following the surgery. Reduction in BMD was most prominent at the femoral neck in patients with a greater reduction in ghrelin concentration.  †Adiponectin appeared to increase in both groups, hinting an inverse correlation with BMD. | | | | | | | | | | | | | | | |
| Miazgowski *et al.*, 2012 ^56^ | 2012 | 57 post-menopausal females newly diagnosed with type-2 diabetes aged 50 to 78 years | Adiponectin | TB  ↓ | | | | | LS (L1-L4)  ↓ | | | TH  ↓ | | | | | | |
|  |  |  | Samples for biochemical assessments were collected at baseline and every 3 months for a 12-month period. BMD measurements were taken at baseline and following 12 months. | | | | | | | | | | | | | | | |
| Sebastián-Ochoa *et al.*, 2012 ^57^ | 2012 | 53 post-menopausal females treated for osteoporosis (33 with alendronate and 20 with raloxifene) with a mean age of 63 ± 7 years | Adiponectin  Leptin | LS  NS | | | FN  NS | | | | | TH  NS | | | | | | |
|  |  |  | This study assessed BMD of the participants prior to treatment and one year following treatment. Adiponectin and leptin levels were also assessed prior and after treatment.  No association was noted between BMD and adiponectin or leptin. | | | | | | | | | | | | | | | |

↓ denotes an observed negative effect on BMD

↑ denotes an observed positive effect on BMD

FN: Femoral neck, FS: Femoral shaft, Intertro: Intertrochanteric, LS: Lumbar spine, NS: non-significant, TB: Total body, TF: Total femur, TH: Total hip, TS: Thoracic spine.

**References**

1. Tariq S, Tariq S, Khaliq S, Lone KP. Serum resistin levels as predictor of low bone mineral density in postmenopausal women. *Health Care Women Int.* 2021;42(1):82-91.

2. Bi X, Loo YT, Henry CJ. Relationships between adiponectin and bone: Sex difference. *Nutrition.* 2020;70:110489.

3. Meng XH, Tan LJ, Xiao HM, Tang BS, Deng HW. Examining the causal role of leptin in bone mineral density: A Mendelian randomization study. *Bone.* 2019;125:25-29.

4. Mihai G, Gasparik AI, Pascanu IM, Cevei M, Hutanu A, Pop RM. The influence of Visfatin, RBP-4 and insulin resistance on bone mineral density in women with treated primary osteoporosis. *Aging Clin Exp Res.* 2019;31(6):889-895.

5. Głogowska-Szeląg J, Kos-Kudła B, Marek B, Nowak M, Siemińska L. Assessment of selected adipocytokines in obese women with postmenopausal osteoporosis. *Endokrynol Pol.* 2019;70(6):478-483.

6. Xu ZH, Zhang X, Xie H, et al. Serum CTRP3 Level is Associated with Osteoporosis in Postmenopausal Women. *Exp Clin Endocrinol Diabetes.* 2018;126(9):559-563.

7. Menzel J, Biemann R, Aleksandrova K, et al. The cross-sectional association between chemerin and bone health in peri/pre and postmenopausal women: results from the EPIC-Potsdam study. *Menopause.* 2018;25(5):574-578.

8. Kadric L, Zylla S, Nauck M, Völzke H, Friedrich N, Hannemann A. Associations Between Plasma Chemerin Concentrations and Bone Quality in Adults From the General Population. *Endocrinology.* 2018;159(6):2378-2385.

9. Chan GMF, Riandini T, Ng SHX, et al. Role of Fat and Bone Biomarkers in the Relationship Between Ethnicity and Bone Mineral Density in Older Men. *Calcif Tissue Int.* 2018;102(1):64-72.

10. Ho-Pham LT, Lai TQ, Nguyen UD, Bui QV, Nguyen TV. Delineating the Relationship Between Leptin, Fat Mass, and Bone Mineral Density: A Mediation Analysis. *Calcif Tissue Int.* 2017;100(1):13-19.

11. Moradi S, Mirzaei K, Abdurahman AA, Keshavarz SA. Adipokines may mediate the relationship between resting metabolic rates and bone mineral densities in obese women. *Osteoporos Int.* 2017;28(5):1619-1629.

12. Tanna N, Patel K, Moore AE, Dulnoan D, Edwards S, Hampson G. The relationship between circulating adiponectin, leptin and vaspin with bone mineral density (BMD), arterial calcification and stiffness: a cross-sectional study in post-menopausal women. *J Endocrinol Invest.* 2017;40(12):1345-1353.

13. Tariq S, Baig M, Tariq S, Shahzad M. Association of serum leptin with bone mineral density in postmenopausal osteoporotic females. *Gynecol Endocrinol.* 2017;33(4):287-291.

14. Haam JH, Kim YS, Kim MJ, et al. A cross-sectional study of the association between adipokine levels and bone mineral density according to obesity and menopausal status in Korean women. *J Bone Miner Metab.* 2017;35(6):642-648.

15. Cervellati C, Bonaccorsi G, Bergamini CM, et al. Association between circulatory levels of adipokines and bone mineral density in postmenopausal women. *Menopause.* 2016;23(9):984-992.

16. Värri M, Niskanen L, Tuomainen T, Honkanen R, Kröger H, Tuppurainen MT. Association of adipokines and estradiol with bone and carotid calcifications in postmenopausal women. *Climacteric.* 2016;19(2):204-211.

17. Mpalaris V, Anagnostis P, Anastasilakis AD, Goulis DG, Doumas A, Iakovou I. Serum leptin, adiponectin and ghrelin concentrations in post-menopausal women: Is there an association with bone mineral density? *Maturitas.* 2016;88:32-36.

18. Menzel J, Di Giuseppe R, Biemann R, et al. Association between omentin-1, adiponectin and bone health under consideration of osteoprotegerin as possible mediator. *J Endocrinol Invest.* 2016;39(11):1347-1355.

19. Zheng T, Yang L, Liu Y, et al. Plasma DPP4 Activities Are Associated With Osteoporosis in Postmenopausal Women With Normal Glucose Tolerance. *J Clin Endocrinol Metab.* 2015;100(10):3862-3870.

20. Aguirre L, Napoli N, Waters D, Qualls C, Villareal DT, Armamento-Villareal R. Increasing adiposity is associated with higher adipokine levels and lower bone mineral density in obese older adults. *J Clin Endocrinol Metab.* 2014;99(9):3290-3297.

21. Li XP, Zeng S, Wang M, Wu XP, Liao EY. Relationships between serum omentin-1, body fat mass and bone mineral density in healthy Chinese male adults in Changsha area. *J Endocrinol Invest.* 2014;37(10):991-1000.

22. Mohiti-Ardekani J, Soleymani-Salehabadi H, Owlia MB, Mohiti A. Relationships between serum adipocyte hormones (adiponectin, leptin, resistin), bone mineral density and bone metabolic markers in osteoporosis patients. *J Bone Miner Metab.* 2014;32(4):400-404.

23. Pedone C, Napoli N, Pozzilli P, et al. Bone Health As a Function of Adipokines and Vitamin D Pattern in Elderly Patients. *Rejuvenation research.* 2013;16.

24. Kocyigit H, Bal S, Atay A, Koseoglu M, Gurgan A. Plasma leptin values in postmenopausal women with osteoporosis. *Bosn J Basic Med Sci.* 2013;13(3):192-196.

25. Morcov C, Vulpoi C, Brănişteanu D. Correlation between adiponectin, leptin, insulin growth factor-1 and bone mineral density in pre and postmenopausal women. *Rev Med Chir Soc Med Nat Iasi.* 2012;116(3):785-789.

26. Tohidi M, Akbarzadeh S, Larijani B, et al. Omentin-1, visfatin and adiponectin levels in relation to bone mineral density in Iranian postmenopausal women. *Bone.* 2012;51(5):876-881.

27. Tenta R, Kontogianni MD, Yiannakouris N. Association between circulating levels of adiponectin and indices of bone mass and bone metabolism in middle-aged post-menopausal women. *J Endocrinol Invest.* 2012;35(3):306-311.

28. Pluskiewicz W, Adamczyk P, Marek B, et al. Adiponectin and resistin in relationship with skeletal status in women from the RAC-OST-POL study. *Endokrynol Pol.* 2012;63(6):427-431.

29. Iida T, Domoto T, Takigawa A, et al. Relationships among blood leptin and adiponectin levels, fat mass, and bone mineral density in Japanese pre- and postmenopausal women. *Hiroshima J Med Sci.* 2011;60(4):71-78.

30. Nakamura Y, Nakano M, Suzuki T, et al. Two adipocytokines, leptin and adiponectin, independently predict osteoporotic fracture risk at different bone sites in postmenopausal women. *Bone.* 2020;137:115404.

31. Menezes AMB, Oliveira PD, Gonçalves H, et al. Are cytokines (IL-6, CRP and adiponectin) associated with bone mineral density in a young adult birth cohort? *BMC Musculoskelet Disord.* 2018;19(1):427.

32. Johansson H, Odén A, Karlsson M, et al. Waning predictive value of serum adiponectin for fracture risk in elderly men: MrOS Sweden. *Osteoporosis International.* 2014;25.

33. Barbour KE, Zmuda JM, Boudreau R, et al. The effects of adiponectin and leptin on changes in bone mineral density. *Osteoporos Int.* 2012;23(6):1699-1710.

34. Johansson H, Odén A, Lerner UH, et al. High serum adiponectin predicts incident fractures in elderly men: Osteoporotic fractures in men (MrOS) Sweden. *J Bone Miner Res.* 2012;27(6):1390-1396.

35. Yan P, Xu Y, Zhang Z, et al. Association of Circulating Omentin-1 with Osteoporosis in a Chinese Type 2 Diabetic Population. *Mediators Inflamm.* 2020;2020:9389720.

36. Legroux-Gérot I, Vignau J, Viltart O, Hardouin P, Chauveau C, Cortet B. Adipokines and bone status in a cohort of anorexic patients. *Joint Bone Spine.* 2019;86(1):95-101.

37. Wu J, Xu J, Wang K, et al. Associations between circulating adipokines and bone mineral density in patients with knee osteoarthritis: a cross-sectional study. *BMC Musculoskelet Disord.* 2018;19(1):16.

38. Huang N, Zhou J, Wang W, et al. Retinol-binding protein 4 is positively associated with bone mineral density in patients with type 2 diabetes and osteopenia or osteoporosis. *Clin Endocrinol (Oxf).* 2018;88(5):659-664.

39. Marchelek-Mysliwiec M, Wisniewska M, Nowosiad-Magda M, et al. Association Between Plasma Concentration of Klotho Protein, Osteocalcin, Leptin, Adiponectin, and Bone Mineral Density in Patients with Chronic Kidney Disease. *Horm Metab Res.* 2018;50(11):816-821.

40. Al-Osami MH, Hameed EK. Serum adiponectin level in osteoporotic postmenopausal women with type 2 diabetes mellitus. *Diabetes Metab Syndr.* 2018;12(6):939-942.

41. Chen DD, Huang JF, Lin QC, Chen GP, Zhao JM. Relationship between serum adiponectin and bone mineral density in male patients with obstructive sleep apnea syndrome. *Sleep Breath.* 2017;21(2):557-564.

42. Gao S, Cheng Y, Zhao L, Chen Y, Liu Y. The relationships of irisin with bone mineral density and body composition in PCOS patients. *Diabetes Metab Res Rev.* 2016;32(4):421-428.

43. Terzoudis S, Malliaraki N, Damilakis J, Dimitriadou DA, Zavos C, Koutroubakis IE. Chemerin, visfatin, and vaspin serum levels in relation to bone mineral density in patients with inflammatory bowel disease. *Eur J Gastroenterol Hepatol.* 2016;28(7):814-819.

44. Sabour H, Norouzi Javidan A, Latifi S, et al. Relationship between leptin and adiponectin concentrations in plasma and femoral and spinal bone mineral density in spinal cord-injured individuals. *Spine J.* 2015;15(1):1-9.

45. Doherty AL, Battaglino RA, Donovan J, et al. Adiponectin is a candidate biomarker of lower extremity bone density in men with chronic spinal cord injury. *J Bone Miner Res.* 2014;29(1):251-259.

46. Ahmadi F, Salari S, Maziar S, Esfahanian F, Khazaeipour Z, Ranjbarnovin N. Relationship between serum leptin levels and bone mineral density and bone metabolic markers in patients on hemodialysis. *Saudi J Kidney Dis Transpl.* 2013;24(1):41-47.

47. Amemiya N, Otsubo S, Iwasa Y, Onuki T, Nitta K. Association between high-molecular-weight adiponectin and bone mineral density in hemodialysis patients. *Clin Exp Nephrol.* 2013;17(3):411-415.

48. Register TC, Divers J, Bowden DW, et al. Relationships between serum adiponectin and bone density, adiposity and calcified atherosclerotic plaque in the African American-Diabetes Heart Study. *J Clin Endocrinol Metab.* 2013;98(5):1916-1922.

49. Okuno S, Ishimura E, Norimine K, et al. Serum adiponectin and bone mineral density in male hemodialysis patients. *Osteoporos Int.* 2012;23(7):2027-2035.

50. Polymeris A, Doumouchtsis K, Grapsa E. Bone mineral density and bone metabolism in hemodialysis patients. Correlation with PTH, 25OHD3 and leptin. *Nefrologia.* 2012;32(1):73-78.

51. Vasilkova O, Mokhort T, Sharshakova T, Hayashida N, Takamura N. Leptin is an independent determinant of bone mineral density in men with type 2 diabetes mellitus. *Acta Diabetol.* 2011;48(4):291-295.

52. Bassatne A, Jafari A, Kassem M, Mantzoros C, Rahme M, El-Hajj Fuleihan G. Delta-like 1 (DLK1) is a possible mediator of vitamin D effects on bone and energy metabolism. *Bone.* 2020;138:115510.

53. Sharma A, Ma Y, Scherzer R, et al. Brief Report: Association of Adipokines With Bone Mineral Density in HIV-Infected and HIV-Uninfected Women. *J Acquir Immune Defic Syndr.* 2016;73(4):433-437.

54. Wang L, Liu L, Pan Z, Zeng Y. Serum leptin, bone mineral density and the healing of long bone fractures in men with spinal cord injury. *Bosn J Basic Med Sci.* 2015;15(4):69-74.

55. Carrasco F, Basfi-Fer K, Rojas P, et al. Changes in bone mineral density after sleeve gastrectomy or gastric bypass: relationships with variations in vitamin D, ghrelin, and adiponectin levels. *Obes Surg.* 2014;24(6):877-884.

56. Miazgowski T, Noworyta-Ziętara M, Safranow K, Ziemak J, Widecka K. Serum adiponectin, bone mineral density and bone turnover markers in post-menopausal women with newly diagnosed Type 2 diabetes: a 12-month follow-up. *Diabet Med.* 2012;29(1):62-69.

57. Sebastián-Ochoa A, Fernández-García D, Reyes-García R, et al. Adiponectin and leptin serum levels in osteoporotic postmenopausal women treated with raloxifene or alendronate. *Menopause.* 2012;19(2):172-177.
